# Extraction and Characterization of Antioxidant Compounds in Almond (*Prunus amygdalus*) Shell Residues for Food Packaging Applications

**DOI:** 10.3390/membranes12080806

**Published:** 2022-08-20

**Authors:** Arantzazu Valdés, María Carmen Garrigós, Alfonso Jiménez

**Affiliations:** Analytical Chemistry, Nutrition & Food Sciences Department, University of Alicante, P.O. Box 99, 03080 Alicante, Spain

**Keywords:** *Prunus amygdalus*, almond, shell residues, microwave-assisted extraction, antioxidant compounds, response surface methodology, food packaging

## Abstract

This work proposes the revalorization of almond shell (AS) wastes as an active additive for food packaging applications. A new microwave-assisted extraction (MAE) method to obtain extracts rich in polyphenolic compounds with high antioxidant capacity was optimized. An experimental design to optimize the MAE procedure through response surface methodology (RSM) using a Box–Behnken design was proposed. The effects of extraction temperature, irradiation time, ethanol:water concentration, and solvent pH at three levels were evaluated in terms of total phenolic content (TPC) and antioxidant activity (DPPH (2,2-diphenyl-1-picrylhydrazyl) and ferric reducing antioxidant power (FRAP) assays). The optimal conditions found were 57 min, 80 °C, pH 8, and 70% (*v*/*v*) ethanol. Optimized MAE extracts showed low soluble protein content (0.43 mg BSA g^−1^) and were rich in TPC (5.64 mg GAE g^−1^), flavonoids (1.42 mg CE g^−1^), and polysaccharides (1.59 mg glucose g^−1^), with good antioxidant capacity (2.82 mg AAE acid g^−1^). These results suggest the potential application of these extracts in the food industry as active additives. This strategy opens new pathways to valorize almond shell residues, contributing to the circular economy.

## 1. Introduction

In recent years, byproduct generation has become a serious concern and waste valorization practices based on circular economy approaches have attracted great attention [1,2]. Almond shell (AS) is the lignocellulosic material of the almond husk, contributing around 35−75 wt% of the total fruit weight [3]. Consequently, around 0.8–1.8 million tons of almond shells are generated annually. Up to now, this byproduct has been used as livestock feed and fuel [4] or it has been incinerated or dumped in landfills, contributing to environmental concerns [1]. Biowaste incineration releases large quantities of greenhouse gases such as methane into the atmosphere. As a consequence, global warming is accelerated, with these gases having an effect 30 times greater than that of CO_2_. Incineration and dumping also contribute to soil and underground water contamination, which may be associated with long-term health effects in exposed communities [5].

Phytochemicals and bioactive compounds have been found in substantial quantities in different vegetable wastes such as seeds, peels, leaves, and stems [6]. These bioactive compounds comprise an excellent pool of molecules with tremendous potential for use as food supplements in food processing or as active additives for food packaging, cosmetics, and pharmaceutical applications [7,8], with several advantages such as availability, recyclability, low cost, environmentally friendly properties, no toxicity, and biodegradability [9]. Several works can be found in the recent literature related to the use of natural antioxidants extracted from agrifood byproducts such as red cabbage, sweet potato, and blue tea [10], among others, for food active packaging applications based on biodegradable materials [11]. These materials can be degraded by the enzymatic action of living organisms such as bacteria, yeasts, and fungi. The use of biomaterials is a sustainable development approach that aids in environmental preservation due to advantages including the materials’ wide availability, nontoxicity, biodegradability, and biocompatibility [12]. Regarding different shell wastes, biodegradable plastic films based on 75% cellulose and 25% fiber bioplastic from cocoa pod husk and sugarcane bagasse wastes have been successfully reported [13]. In a different work, cellulose nanocrystals isolated from water hyacinth fiber were studied as a reinforcement for a polyvinyl alcohol (PVA)–gelatin nanocomposite [14]. Thus, the development of novel biodegradable materials with functional properties is an emerging research interest.

In particular, AS has been previously reported to be rich in bioactive compounds such as triterpenoids (betulinic, urosolic, and oleanolic acids), cinnamic acid derivatives (caftaric and chlorogenic acids), flavonol glycosides (kaempferol and quercetin glycosides and aglycones), flavan-3-ols (represented by catechin and epicatechin), and flavanone derivatives including naringenin and isorhamnetin derivatives [4,15,16]. As a result, previous investigations on AS have shown interesting antioxidant and antiradical properties [17,18].

Different techniques for recovering antioxidant compounds from vegetable matrices can be used, with traditional extraction techniques, such as simple agitation or maceration, showing several disadvantages. On one hand, they generally require high solvent consumption and are long-duration procedures. In addition, their operation at high temperatures could create a risk of thermal degradation for most of the bioactive compounds [19]. As an alternative, environmentally friendly techniques have been developed to overcome these drawbacks in recent years, including ultrasound-assisted extraction, pressurized liquid extraction, microwave-assisted extraction, supercritical fluid extraction, and enzyme-assisted extraction [20]. In this work, MAE was proposed as a green, efficient technology for the extraction of antioxidants from AS waste, requiring lesser quantities of organic solvents, reducing extraction time and energetic requirements, and leading to high extraction yields [21,22,23]. The most commonly reported techniques used for antioxidant extraction from AS have been solvent extraction [15,18] and maceration [17]. However, few studies have been reported in the literature regarding the use of greener and more advanced techniques. In this sense, only one related study was found, in which ultrasound-assisted extraction was used to obtain antioxidant compounds from AS [4]. MAE has only been reported for the extraction of almond skin [24] and no studies regarding almond shell wastes were found.

This work proposes the revalorization of AS wastes as an active additive for food packaging applications. Thus, a new MAE method to obtain extracts rich in polyphenolic compounds with high antioxidant capacity from AS wastes was optimized via response surface methodology (RSM) using a Box–Behnken design (BBD). These statistical tools for process optimization show several advantages such as reduction in experimental runs, time consumption, and costs, allowing relevant data to be obtained from the analyses without reducing their quality [25,26,27]. The obtained extracts were characterized in terms of antioxidant performance and total polyphenol (TPC), flavonoid, soluble protein, and polysaccharide contents to assess their potential as active additives in the food industry, with the valorization of almond shell wastes providing added value to almond production residues and reducing environmental impact.

## 2. Materials and Methods

### 2.1. Materials and Reagents

**Figure 1:**
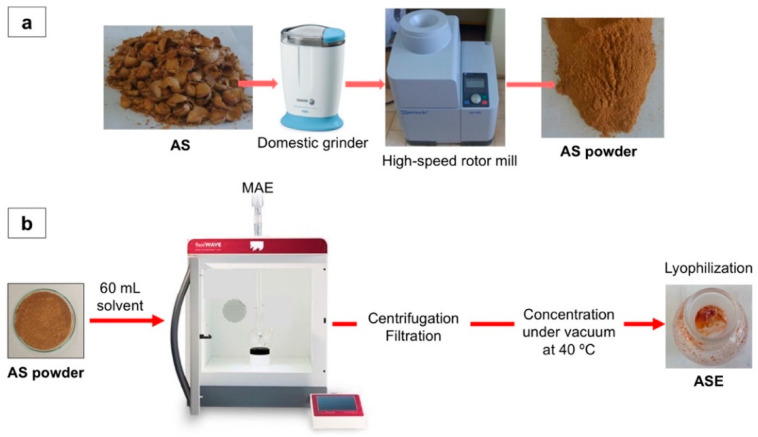
Scheme of (**a**) AS powder preparation and (**b**) MAE process to obtain ASE.

AS industrial wastes from the Marcona almond cultivar were obtained from Sirvent Almendras S.A. (Alicante, Spain). First, 100 g of AS was washed with 500 mL of cold distilled water followed by drying at ambient temperature for 12 h and further drying at 40 °C for 4 h. A fine AS powder was obtained through two consecutive grinding steps (Figure 1a). Firstly, AS was placed in a domestic grinder (Fagor, Spain) at 300 rpm for 15 s to reduce its initial size, and then a fine powder was obtained with a high-speed rotor mill (Ultra Centrifugal Mill ZM 200, RETSCH, Haan, Germany) equipped with a 1 mm sieve size at a maximum peripheral rotor speed of 92 m s^−1^. All chemicals and reagents were of analytical grade and were purchased from Sigma-Aldrich (Madrid, Spain).

### 2.2. Microwave-Assisted Extraction (MAE)

MAE was performed using a FLEXIWAVE^TM^ microwave oven (Milestone srl, Bergamo, Italy). First, 4.000 ± 0.001 g of homogenized AS powder was placed in a 100 mL quartz flask connected to a vapor condenser and containing 60 mL of the extraction solvent. This AS/solvent ratio was selected according to previous tests in order to use a high quantity of sample without the formation of almond shell aggregates in the quartz flask during the extraction phase. Samples were stirred at 500 rpm and heated to the desired temperature for selected extraction times, allowing a homogeneous heating. The obtained extracts were centrifuged at 4500 rpm for 5 min and then filtered through a 0.45 μm PVDF filter (Teknokroma, Barcelona, Spain). After filtration, the solvent was removed under vacuum at 40 °C in a rotary evaporator (R-300, Büchi Labortechnik AG, Flawil, Switzerland), followed by lyophilization (LyoQuest Plus, Telstar, Madrid, Spain) to obtain the dried AS extract (ASE) (Figure 1b). Before all analyses described in this work, dried ASE samples were made up to 12 mL in deionized water. 

### 2.3. MAE Optimization

RSM was used to determine the optimal extraction conditions of antioxidant compounds from AS by MAE. A BBD, comprising 29 experimental runs, was used. Five central points were added to evaluate the experimental error. Tests were carried out in a randomized order to evaluate the effects of four factors at three levels: extraction temperature (40, 60, 80 °C), irradiation time (15, 37.5, 60 min), ethanol:water concentration (40, 60, 80%, *v*/*v*), and solvent pH (2, 7, 12). HCl (1 mol L^−1^) was used to adjust pH = 2, whereas pH 7 and 12 were adjusted with phosphate buffer (pH = 7) and NaOH (1 mol L^−1^), respectively. The levels of the experimental design were set according to the reported bibliography and experimental limitations. The responses obtained from the experimental design were evaluated in terms of total phenolic content (TPC) and antioxidant activity determined using the DPPH (2,2-diphenyl-1-picrylhydrazyl) and ferric reducing antioxidant power (FRAP) assays (Table 1).

Regression analysis was used for the experimental data, which were fitted using the following empirical second-order polynomial model (Equation (1)):

Y = β_0_ + Σβ_i_X_i_ + Σβ_ii_X_i_ + ΣΣβ_ij_X_i_X_j_ (1)

where Y is the predicted response; X represents the variables of the system; i and j are the design variables; β_0_ is a constant; β_i_ and β_ii_ are the linear and quadratic coefficients, respectively; and β_ij_ is the interaction coefficient of variables i and j. The adequacy of the model was determined by evaluating the coefficient of determination (R^2^), adjusted R_2_, lack of fit, and coefficients of variation (CV) obtained from the analysis of variance (ANOVA). Statistical significance of the model and model parameters was determined at the 5% probability level (α = 0.05).

### 2.4. Characterization of AS Extracts

#### 2.4.1. Antioxidant Activity

The DPPH assay was used to determine the free radical scavenging activity of ASE [24]. Trolox was used as standard and results were expressed as mg of Trolox equivalents (TE) g AS^−1^ (DW). A calibration curve obtained with Trolox in the range of 25–275 mg kg^−1^ (R^2^ = 0.9690) was used for the DPPH assay following a previous work [22]. The IC_50_ value was calculated at 517 nm, in triplicate, as the concentration of ASE required to quench 50% of the initial DPPH radical by using 175 mg of extract per mL to prepare the dilution solutions.

The capacity of ASE to reduce ferric ions was assessed by using the FRAP method according to a previous work with some modifications [24]. First, 50 µL of ASE was added to 1.5 mL of FRAP reagent. Measurements were performed at 593 nm using a Biomate-3 spectrophotometer (Thermospectronic, Mobile, AL, USA), in triplicate, after 30 min of incubation at 37 °C in darkness. Ascorbic acid was used as the standard (2–78 mg kg^−1^, R^2^ = 0.9998) and the antioxidant capacity was expressed as mg of ascorbic acid equivalents (AAE) per gram of AS (DW).

The ABTS (2, 2′ azinobis (3-ethylbenzthiazoline)-6-sulfonic acid) free radical decolorization assay was performed as reported elsewhere with some modifications [28]. First, 10 μL of ASE was mixed with 1 mL of the ABTS solution. A calibration curve was prepared using Trolox (150–750 mg kg^−1^, R^2^ = 0.9969) and results were expressed as mg of Trolox equivalents (TE) g AS^−1^ (DW). Tests were performed in triplicate.

#### 2.4.2. Total Phenolic Content

The Folin–Ciocalteu method was carried out according to Lin et al. [29]. An aliquot (50 µL) of ASE was mixed with 1 mL of deionized water and 0.5 mL of Folin–Ciocalteu’s phenol reagent. Next, 2.5 mL of 10% Na_2_CO_3_ solution was added, followed by an incubation period of 20 min in the dark. The absorbance was recorded at 735 nm with a Biomate-3 spectrophotometer (Thermospectronic, Mobile, AL, USA). A standard curve using gallic acid was prepared (25–1005 mg kg^−1^, R^2^ = 0.9997) and results were expressed as mg gallic acid equivalents (GAE) g AS^−1^ (DW). Tests were performed in triplicate.

#### 2.4.3. Extraction Yield

The extraction yield (%) was determined, in triplicate, following Equation (2), where W is the weight of the dried ASE and W_0_ is the weight of AS powder.

Extraction yield= W/W_0_ ∗ 100 (2)

#### 2.4.4. Flavonoid Content

Flavonoid content was determined according to Lin et al. [29]. First, 250 µL of ASE was mixed with 1.25 mL of 165 deionized water. Then, 75 µL of 5% NaNO_2_ solution was added. After 6 min, 150 µL of 10% AlCl_3_.H_2_O solution was added, followed by reaction for 5 min. After the addition of 1 M NaOH solution (0.5 mL) and 275 µL of ethanol, the absorbance was read at 510 nm using a Biomate-3 spectrophotometer (Thermospectronic, Mobile, AL, USA). A standard curve using catechin was used (27–2117 mg kg^−1^, R^2^ = 0.9984) and results were expressed as mg catechin equivalents (CE) per gram of AS, DW. Tests were performed in triplicate.

#### 2.4.5. Soluble Protein Content

The Bradford method using bovine serum albumin (BSA) was used in this study, as previously described for almond nuts [30,31]. The dyeing solution for protein determination was prepared by diluting 12 mg of Comassie blue brilliant G-250 (CBBG, purchased from Merck) in 10 mL absolute ethanol with 3% perchloric acid solution to 200 mL. Next, 1 mL of CBBG solution and an aliquot of AS extract were mixed in a cuvette and completed to 3 mL with buffer pH 7. The absorbance was measured, in triplicate, at 596 nm with a Biomate-3 spectrophotometer (Thermospectronic, Mobile, AL, USA). BSA was used as a reference water-soluble protein for the calibration curve (10–995 mg Kg^−1^, R^2^ = 0.9991) and results were expressed as mg of protein per gram of AS (DW).

#### 2.4.6. Total Polysaccharide Content

The phenol–sulfuric acid method was used to obtain the total polysaccharide content [28]. A 2 mL aliquot of an ASE was mixed with 1 mL of a 5% aqueous solution of phenol (obtained from Fischer Scientific, Waltham, MA, USA). Subsequently, 5 mL of concentrated H_2_SO_4_ was rapidly added to the mixture. After the test tubes were allowed to stand for 10 min, they were vortexed for 30 s and placed for 20 min in a water bath at room temperature for color development. Absorbance was then measured in triplicate at 490 nm using a Biomate-3 spectrophotometer (Thermospectronic, Mobile, AL, USA). Glucose was used as the reference for the calibration curve (20–219 mg Kg^−1^, R^2^ = 0.9949) and results were expressed as mg of glucose per gram of AS (DW). 

#### 2.4.7. Morphological Analysis

The morphological structure of AS and dried ASE was analyzed using a JEOL JSM-840 scanning electron microscope (Peabody, MA, USA) under an acceleration voltage of 15 kV. Prior to scanning, a SCD 004 Balzers sputter coater (Bal Tec. AG, Fürstentum, Lichtenstein) was used in order to coat samples with a gold layer under vacuum. Images were registered at magnifications of 500, 1000, and 2500×.

### 2.5. Statistical Analysis

Statgraphics-Plus software 5.1 (Statistical Graphics, Rockville, MD, USA) was used to generate and analyze the results of the BBD. All results are expressed as mean values ± standard deviation (SD). SPSS commercial software, ver. 15.0 (Chicago, IL, USA), was used for ANOVA analysis. Tukey test at a *p* ≤ 0.05 significance level was assessed to obtain differences between values. 

## 3. Results and Discussion

### 3.1. Fitting the Models

Multiple regression analysis was applied to the experimental data (Table 1), with all the studied responses (DPPH, FRAP, and TPC) being expressed as a function of the independent variables (extraction temperature, ethanol concentration, pH, and irradiation time), resulting in second-order polynomial equations. The reliability of the fitted models and the statistical significance of the regression coefficients were studied by performing an analysis of variance (ANOVA) [32]. The second-order polynomial equations obtained for the three responses were expressed as follows:

DPPH = −1.17188 + 0.0526259 ∗ A + 0.00912769 ∗ B + 0.206608 ∗ C + 0.00427031 ∗ D − 0.000470702 ∗ A^2^ + 0.000294677 ∗ A ∗ B + 0.000199397 ∗ A ∗ C −    0.0000259385 ∗ A ∗ D − 0.000300107 ∗ B^2^ + 0.000262823 ∗ B ∗ C + 0.000219005 ∗ B ∗ D − 0.0200391 ∗ C^2^ − 0.0000210329 ∗ C ∗ D − 0.00017416 ∗ D^2^      (3)

FRAP = −0.609436 + 0.0535699 ∗ A − 0.0541801 ∗ B + 0.197749 ∗ C + 0.0127485  ∗ D − 0.000689 ∗ A^2^ + 0.000198274 ∗ A ∗ B + 0.00247619 ∗ A ∗ C + 0.00079343 ∗ A ∗ D + 0.00027336 ∗ B^2^ + 0.003072 ∗ B ∗ C − 0.00010136 ∗ B ∗ D − 0.0274489 ∗ C^2^ + 0.0009920 ∗ C ∗ D − 0.0007529 ∗ D^2^                 (4)

TPC = −7.00819 + 0.0134883 ∗ A + 0.189039 ∗ B + 0.687649 ∗ C − 0.00198112 ∗  D − 0.00001184 ∗ A^2^ + 0.0001335 ∗ A ∗ B − 0.000396 ∗ A ∗ C + 0.0015187 ∗ A ∗ D − 0.001335 ∗ B^2^ − 0.0064199 ∗ B ∗ C − 0.0005226 ∗ B ∗ D − 0.0230505 ∗ C^2^ + 0.001150 ∗ C ∗ D − 0.0006496 ∗ D^2^                    (5)

where A, B, C, and D represent extraction temperature, ethanol concentration, pH and irradiation time, respectively.

The adequacy of the model was determined by evaluating the model fitting parameters from the ANOVA, shown in Table 2. The elevated *p*-values (0.7296, 0.1696, and 0.2726 for DPPH, FRAP, and TPC, respectively) indicated the nonsignificance of the lack of fit and the goodness of fit of the models to predict the studied responses. The obtained R^2^ values for DPPH, FRAP, and TPC (0.8547, 0.8119, and 0.9269, respectively, which were quite close to the adjusted R^2^ values) as well as CV values ranging from 9.18 to 16.68%, showed the adequacy of the correlation between the model and experimental data and suggested good precision of the models. In conclusion, a relatively high degree of correlation between experimental data and predicted values was obtained, indicating that all models could be used to predict the studied responses and confirming the reliability of the fitted models. 

### 3.2. Optimal MAE Conditions

The three studied response variables were simultaneously optimized by using a desirability function in order to maximize them. As a result, the optimal operating conditions were: 80 °C, 70% (*v*/*v*) ethanol, pH 8, and 57 min (desirability value of 0.823). Under these conditions, the response values predicted by the models were 1.90 mg TE g^−1^ AS (DW), 3.01 mg AAE g^−1^ AS (DW), and 5.61 mg GAE g^−1^ AS (DW) for DPPH, FRAP, and TPC, respectively. In order to verify the models, three experiments were performed under optimal conditions with experimental values of 1.05 ± 0.05 mg TE g^−1^ AS (DPPH), 2.82 ± 0.08 mg AAE g^−1^ AS (FRAP), and 5.64 ± 0.06 mg GAE g^−1^ AS (TPC); all of these values did not differ significantly from the predicted values (Table 3). Thus, it can be concluded that the obtained quadratic models for DPPH, FRAP, and TPC were reliable for the MAE optimization of antioxidant compounds from AS.

Different extraction procedures for AS have been previously described in the literature. In a previous study, 30 g measures of Fascionello, Pizzuta, and Romana Sicilian cultivars were extracted by maceration using 100 mL of solvent (ethanol:water, 1:1, *v*/*v*) three times for three days [17]. In a different study, AS samples were subjected to extraction in a rotary shaker for 90 min at 50 °C using methanol [18]. Conventional extraction was also carried out with 10 g of AS powder mixed with 100 mL of ethanol:water solution (70/30, *v*/*v*) at 50 °C for 6 h with an orbital shaker [4]. In another work, the extraction of 50 mg of AS was optimized using an orbital shaker and a Box–Behnken design, with optimal conditions for obtaining the highest antioxidant activity and TPC values being 2 mL of 63% ethanol at pH 1.5 for 235 min [15]. Although it is difficult to compare MAE procedures with other extraction methodologies due to the use of different extraction equipment and parameters, it can be concluded that, in general terms, the use of MAE to extract antioxidant compounds from AS significantly reduces the extraction time, i.e., from 6 h or 90 min to less than 60 min. In addition, MAE avoids the use of high temperatures for long irradiation times, which may increase the degradation of labile target compounds at high temperatures [4]. The high efficiency of MAE can be attributed mainly to two phenomena. On one hand, dipole rotation and ionic conduction allow friction or motion between the ions or molecules, and, consequently, electromagnetic waves are transformed into thermal energy [20]. As a result, the temperature in food matrices rises with the disruption of the cell wall structure, increasing the release of phenolic compounds and obtaining high recoveries. On the other hand, pressure increases inside the sample container, modifying the physical properties of the biological tissues, improving the porosity of the biological matrix and allowing a better penetration of the extracting solvent through the matrix [33,34]. As a result, MAE could potentially reduce procedure costs when applied at industrial scale, increasing environmental sustainability. 

### 3.3. Effect of Extraction Variables on Antioxidant Capacity (DPPH and FRAP)

**Figure 2:**
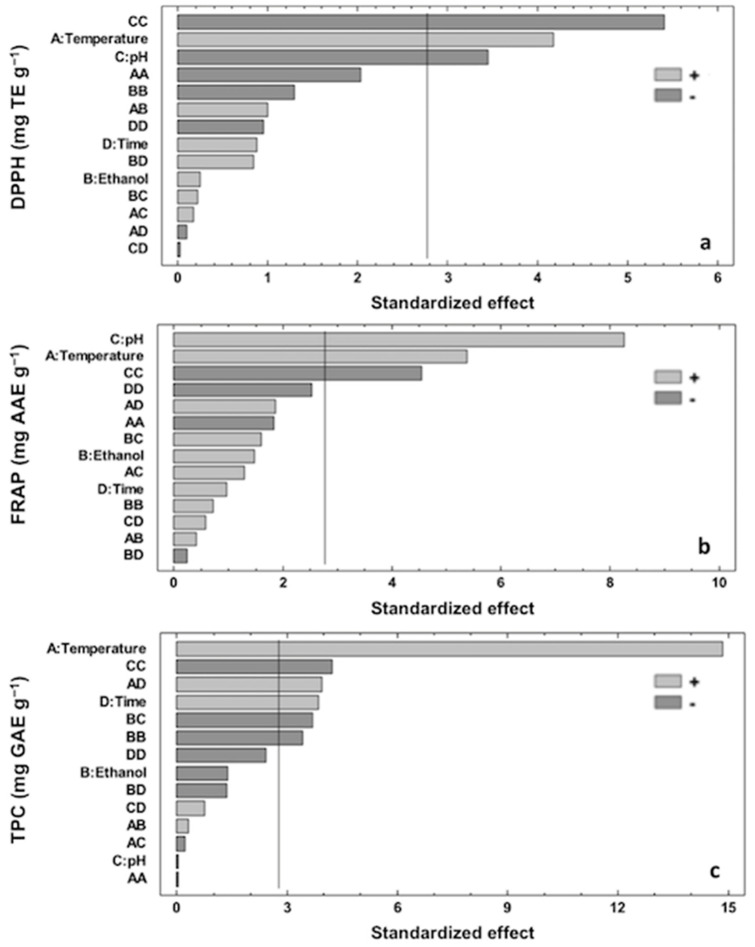
Pareto charts obtained for (**a**) DPPH (mg TE g^−1^), (**b**) FRAP (mg AAE g^−1^), and (**c**) TPC (mg GAE g^−1^) values of ASE by MAE. The vertical line indicates the statistical significance at 5% of the effects.

The Pareto chart and significant effects (95% confidence) obtained from the BBD for DPPH response are shown in Figure 2a. DPPH values obtained from ASE ranged from 0.42 to 2.04 mg TE per g of AS (Table 1). As can be observed in the Pareto chart, DPPH was significantly influenced by temperature (A) and pH (C, C^2^), with the rest of the investigated parameters showing no significant impact on the studied response (*p* > 0.05). The influence of the different significant variables on DPPH was in the following order: C^2^ > A > C. A positive effect was observed for A, whereas C^2^ and C showed a negative effect on DPPH response.

According to the Pareto chart, the most significant negative effect of C^2^ on DPPH indicated that the antioxidant activity of ASE was very sensitive to pH changes in the solution. This behavior was in accordance with several studies underlining the fact that different pH values can affect the stability and antioxidant activity of phenolic extracts [35,36,37,38]. The second factor with a significant impact on DPPH was the extraction temperature, which showed a positive effect on the extraction of antioxidant compounds, in agreement with previous studies dealing with the optimization of antioxidant extracts from several vegetable matrices such as Quercus bark [34], carob bark [39], cocoa bean shell [28], and eggplant peel [22].

**Figure 3:**
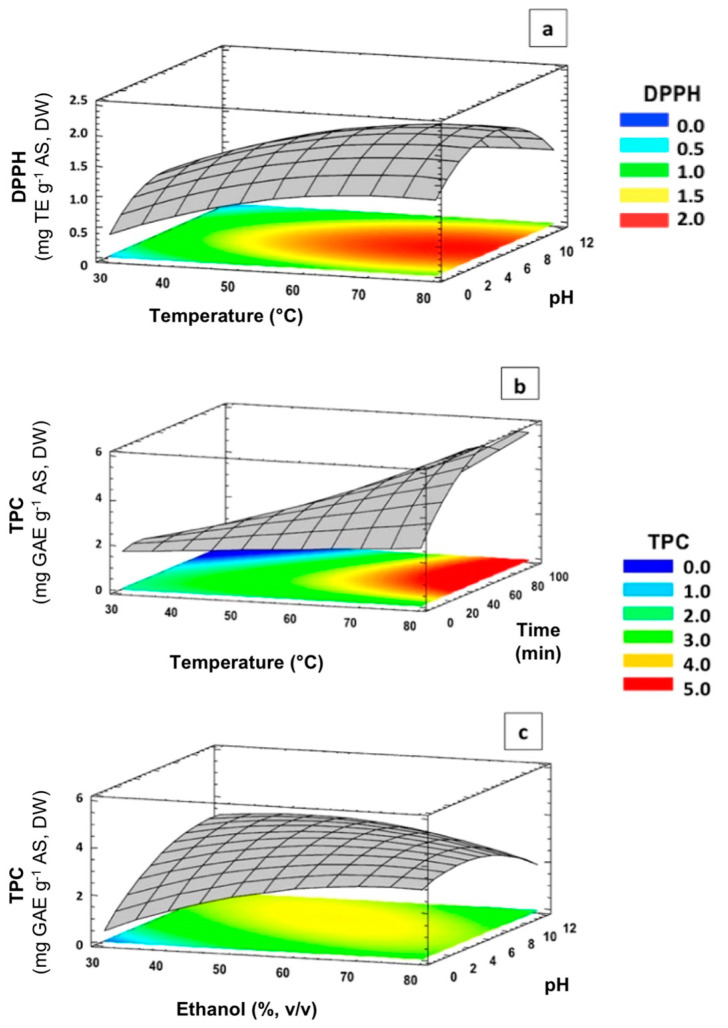
Response surface and contour plots showing interactions on DPPH ((**a**): temperature vs. pH) and TPC ((**b**): temperature vs. time; (**c**): ethanol concentration vs. pH) values of ASE by MAE.

A graphical analysis conducted in order to study the interactions of independent variables on DPPH in terms of response surface and contour plots is shown in Figure 3a. The antioxidant capacity as determined by DPPH was improved by increasing temperature at neutral or slightly alkaline pH values. Natural fibers consist of cellulose and other noncellulose constituents, such as lignin, hemicellulose, pectin, and extractives, composed of wax, proteins, ash, and oil [40]. Specially, Marcona AS has been reported to be composed of 53% of lignin followed by 41% α-cellulose, 11% moisture, 9% hemicellulose, and 0.7% extractives [41]. Different works have underlined that under acid [42] or alkaline conditions [40], the denaturation of cell walls could increase. Thus, the molecular movement from the cells towards the solvent could be sped up, promoting the concentration of metabolites into the solution. Regarding temperature, it is well known that increasing temperature results in higher mass transfer due to the added energy and the decrease in solvent viscosity [43]. Based on the results found in our work, it is suggested that high temperatures combined with acid or highly alkaline media decreased the antioxidant activity of ASE. This fact could be explained by considering that the destruction of the cells of AS tissues could increase the release of active substances into the solvent, increasing both antioxidant and nonantioxidant compounds [36]. Therefore, the composition of AS extracts obtained at pH values of 2 or 12 could be affected along with, consequently, their antioxidant activity [24]. In this line, Zeynep et al. reported that an acid pH value during ultrasound-assisted extraction of polyphenols from mandarin supports the cleavage of phenolics bonded to proteins and carbohydrate polymers [44]. Rubio-Senent et al. studied the influence of two different pH values (4.5 and 2.5) on the composition and antioxidant characteristics of phenolic extracts obtained from an olive oil byproduct (alperujo) with ethyl acetate extraction, showing that the extract obtained at pH 2.5 had higher amounts of sugar degradation products [36]. Mellinas et al. found a greater efficiency in the extraction of proteins, polyphenols, and polysaccharides from cocoa bean shells by MAE at high pH values, contributing to an increased overall extraction yield [28]. Thus, it can be concluded that the composition of extracts could affect also their antioxidant activity, with this being not only dependent on their polyphenolic content [45].

Regarding FRAP results, the values obtained from ASE ranged from 0.27 to 4.15 mg AAE per g of AS (Table 1). The obtained Pareto chart (Figure 2b) underlined that FRAP response was also significantly influenced by temperature (A) and pH (C, C^2^), with the rest of the investigated parameters not showing significant impacts on the studied response (*p* > 0.05). The influence of the different significant variables on FRAP was in the following order: C > A > C^2^. A similar trend to that discussed for DPPH values was found, with a positive effect observed for A and a negative effect for C^2^, whereas C showed a positive effect on FRAP response. The positive influence of temperature and the negative quadratic effect of pH on this antioxidant activity response should be similar to those already discussed for DPPH.

### 3.4. Effect of Extraction Variables on TPC

The TPC values of AS extracts obtained under tested MAE conditions ranged from 1.51 to 6.25 mg GAE per g of AS (Table 1). These results are in accordance with previous works evaluating TPC values obtained after ultrasound-assisted extraction [4] and maceration [17] of almond hulls using ethanol as a solvent. As can be seen in Figure 2c, TPC was significantly influenced by temperature (A) and time (D) as well as ethanol concentration and pH with a quadratic effect (B^2^ and C^2^, respectively). Two significant interactions between temperature and time (AD) and ethanol concentration vs. pH (BC) were also observed. The rest of the investigated variables had no significant effect on the studied response (*p* > 0.05). The influence of the different significant variables on the total polyphenol content followed the order: A > C^2^ > AD > D > BC > B^2^. Positive effects were observed for A, AD, and D parameters, whereas C^2^, BC, and B^2^ showed negative effects on TPC response. The effects of significant interactions of independent variables on TPC response are shown in Figure 3b,c.

**Figure 4:**
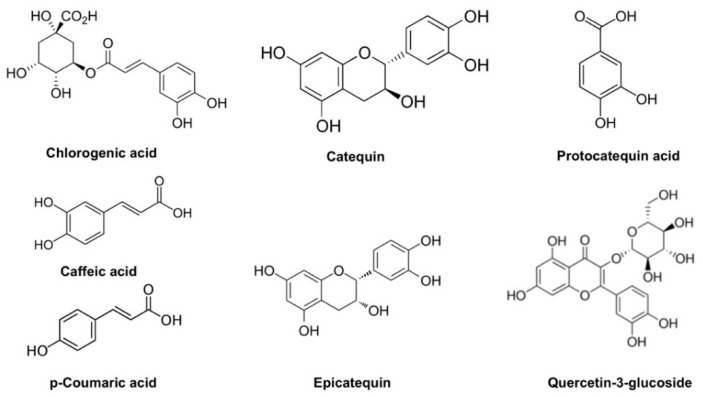
Chemical structures of main polyphenolic compounds reported in AS residues.

The positive influence of temperature and the negative quadratic effect of pH on TPC were in agreement with the behavior found for antioxidant activity (DPPH and FRAP). The ionization of phenolic compounds present in ASE in ethanolic medium could be affected by pH conditions, with this effect being mainly associated with the large number of hydrogen acceptor and donor sites on the polyphenol molecules [46]. The antioxidant capacity of AS could be related to its phenolic compound content, with the most abundant polyphenols, according to previous reported works, being chlorogenic acid and catequin followed by protocatequin acid, caffeic acid, epicatechin, p-coumaric acid, and quercetin-3-glucoside [4]. As shown in Figure 4, a high average number of phenolic-OH groups can be found in this group of compounds, with potential hydrogen-atom transfer sites which can be related to antioxidant capacity.

Regarding irradiation time, a positive influence on TPC response was observed when combined with high temperatures (Figure 3b) which can be mainly explained by considering the thermal accumulation with increasing temperature within the extraction solvent due to the MAE energy over long times. As a consequence, the dissolution of phenolic compounds could take place into the solution. Similar results were reported for TPC optimization by MAE from almond skin residues [24], showing no significant decrease in TPC values under high time and temperature extraction conditions. This fact could suggest that the extracted polyphenols were not degraded by MAE at the studied temperatures.

Concerning ethanol concentration in the solvent extraction, Figure 2c shows the negative quadratic effect of this variable on TPC response. As previously reported, the extraction efficiency depends on the solubility of the analytes in the extraction solvent [47]. Hughey et al. [48] studied the distribution of polyphenols from almond skin in water and ethanol solvents as a function of time and temperature. From this study, the authors concluded that the highest extraction yields of phenolic compounds were achieved as the ethanol fraction increased. However, the use of high ethanol concentrations could lead to polyphenol degradation due to the overheating of the sample and overpressure inside the vessel as a result of dipolar rotation and ionic conduction during the MAE [23]. As a consequence, a faster rate of solvent heating with regards to the plant material is carried out due to the higher capacity of the solvent to absorb microwave energy [24]. In addition, a decrease in ethanol concentration should increase the dielectric constant of the system due to the higher water content, which could increase microwave adsorption and temperature inside the sample as well as improving the swelling of the food material, resulting in the rupture of cells and an increase in surface area of contact between the matrix and the solvent, respectively [39].

Finally, it is interesting to underline the negative significant interaction between ethanol concentration and pH on TPC response (Figure 2c). As can be seen, the obtained results revealed that the highest TPC values could be obtained at slightly alkaline pH values and ethanol concentrations of 60–70% (*v*/*v*) (Figure 3c). Librán et al. (2013) reported that the influence of solvent pH on TPC values cannot be considered independently, but must be combined with ethanol concentrations [45]. According to our findings and the available literature, it can be suggested that pH, ethanol concentration, extraction temperature, and irradiation time are all significant parameters to be considered in the extractability of polyphenolic compounds by MAE and overall antioxidant activity of ASE [21,34,49].

### 3.5. Chemical Characterization of ASE Obtained under Optimal Conditions

The optimized MAE conditions allowed AS extracts to be obtained with an extraction yield of 35.2 ± 0.07 wt% with low contents of soluble proteins (0.43 ± 0.08 mg BSA g^−1^) and total polysaccharides (1.59 ± 0.05 mg glucose g^−1^) (Table 3). The flavonoid content of optimized ASE was 1.42 ± 0.05 mg CE per gram of AS, which is in accordance with values obtained for AS extracts from the Zahaf cultivar, ranging from 0.87 to 3.08 mg CE per gram of AS when using ethanol/water solutions and conventional solvent extraction or ultrasound-assisted extraction, respectively [4]. In a different study, a BBD was used to optimize the flavonoid content of AS when using an orbital shaker with different ethanol concentrations (30, 60, and 90%), with results ranging from 1.74 to 6.05 mg CE per gram of sample. According to these authors, the AS extracts were mainly composed of proanthocyanins and oligomeric flavonoids [15]. These compounds consist of two or more aromatic rings with one hydroxyl group.

The antioxidant activity determined by ABTS, DPPH, and FRAP assays underlined the antioxidant activity of AS (Table 3). Pinelo et al. studied the RSA of AS in ethanolic, methanolic, and water extracts by using a rotary shaker according to a full factorial 23 experimental design. The best results were obtained at 90 min, 50 °C, and a 5:1 liquid–solid ratio with an RSA value of 36% when 96% ethanol was used as extraction solvent. The obtained results in our work demonstrate the superior capacity of the MAE technique to obtain AS extracts with a high capacity to scavenge free radicals (78%, Table 3). This fact was also corroborated by the IC_50_ value obtained by the DPPH assay of 64.96 ± 1.17 mg mL^−1^.

### 3.6. Morphological Characterization of Optimized ASE

**Figure 5:**
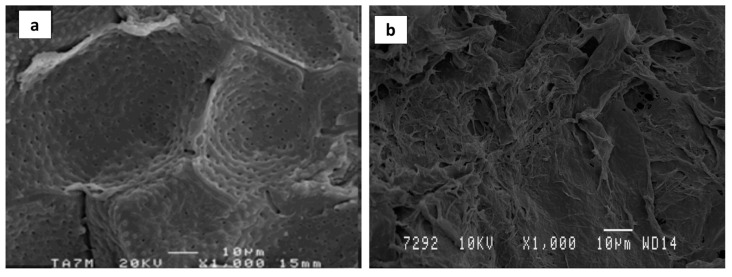
SEM micrographs of AS before (**a**) and after MAE under optimal conditions (**b**) at 1000×.

The surface morphology of AS before and after MAE optimal conditions was investigated using SEM (Figure 5). AS has been reported to be rich in lignin and other minor noncellulosic compounds such as ashes, extractives, and hemicellulose. Hemicelluloses act as connectors between cellulose and lignin. As a consequence, untreated AS (Figure 5a) showed a continuous, smooth, and hard structure [50,51]. Regarding AS SEM micrographs after MAE (Figure 5b), significant changes were observed in the surface morphology which could be related to the partial disruption of the hard AS structure due to the removal of lignin, hemicellulose, and other minor compounds by the action of the alkali medium and the effect of microwave irradiation, allowing the faster release of active compounds from the AS matrix [24,41,52].

## 4. Conclusions

MAE has been shown to be a high potential technique that can be combined with RSM methodology to recover antioxidant compounds from AS, obtaining valuable information about the extraction process. The present study reflects the importance of controlling the extraction temperature, ethanol concentration, pH, and irradiation time to obtain an extract rich in TPC with good antioxidant activity. The optimal conditions to simultaneously maximize TPC and antioxidant activity on ASE were as follows: 57 min of irradiation time, 80 °C of extraction temperature, pH 8, and 70% (*v*/*v*) ethanol content. In contrast to different conventional protocols reported for antioxidant compound extraction in AS, MAE shows some advantages: (a) the procedure can be performed in the dark with absence of light, (b) short extraction times (less than 1 h), (c) the use of closed systems reduces the risk of accidental losses, (d) solvent consumption is considerably reduced, and (e) this technique combines temperature and pressure with the simultaneous breakdown of the cell walls. As a result, MAE could decrease process costs and increase environmental sustainability when used as an extraction technique, obtaining high yields of target compounds. In conclusion, the green methodology proposed in this work has shown great potential for the valorization of almond shell residues in the food industry within a circular economy approach.

The chemical characterization of ASE obtained under optimal conditions underlined its noticeable capacity to scavenge free radicals, linked with a high total phenolic content. For this reason, it is proposed for use in antioxidant packaging as a very promising alternative for extending food product shelf-life. Nowadays, antioxidant food packaging options used in industry mainly include vacuum or air conditioning packaging with a reduction of oxygen concentration to reduce food decomposition. They usually include iron-based, organic- and inorganic-based, polymer-based, and enzyme-based scavenging systems. Although they show several advantages such as low cost, simple process, and suitability for mass production, they usually are present with a small bag visible inside the food container which could cause rejection by the consumer, as they are substances that are not always in line with the new circular economy concept. The developed ASE presents possibilities for use directly as a coating or by introducing it as an active agent into different polymeric matrices, avoiding additional devices or even preservatives in food and reducing the final price as well as the material wastes generated from packaging material, while also being an interesting approach to revalorize AS by-products. Further work will be needed to evaluate the antioxidant properties of the obtained active films when directly applied to food products. Also, including industrial processing extrusion techniques could be interesting in order to obtain commercial products based on these formulations. 

## Figures and Tables

**Table 1:** Box–Behnken experimental design matrix and DPPH, FRAP, and TPC results, expressed on a dry-weight (DW) basis, for ASE by MAE.

Run	Temperature (°C)	Ethanol (%, *v*/*v*)	pH	Time (min)	DPPH (mg TE g^−1^)	FRAP (mg AAE g^−1^)	TPC (mg GAE g^−1^)
1	60	60	7	37.5	1.57	2.28	3.68
2	60	60	12	60.0	1.01	1.78	2.97
3	60	60	7	37.5	1.57	1.66	3.25
4	40	60	2	37.5	0.85	0.27	1.51
5	60	80	2	37.5	1.42	0.54	2.96
6	60	60	7	37.5	1.97	2.01	3.93
7	80	80	7	37.5	1.77	2.58	5.07
8	60	80	7	15.0	1.51	1.71	2.97
9	40	60	7	15.0	1.32	1.25	2.15
10	60	60	2	15.0	1.42	0.38	2.94
11	60	80	12	37.5	1.09	4.15	1.52
12	60	40	7	15.0	1.68	1.53	2.30
13	40	60	7	60.0	1.42	0.81	1.99
14	60	80	7	60.0	1.76	1.82	3.12
15	40	40	7	37.5	1.46	1.32	1.57
16	80	40	7	37.5	1.54	2.38	4.85
17	40	80	7	37.5	1.21	1.21	1.58
18	60	40	7	60.0	1.54	1.82	3.40
19	60	40	2	37.5	1.38	0.31	2.81
20	60	60	7	37.5	1.96	2.23	3.84
21	80	60	7	15.0	1.71	2.16	3.67
22	40	60	12	37.5	0.42	1.04	2.17
23	60	60	12	15.0	0.79	1.39	2.24
24	60	60	2	60.0	1.64	0.32	3.16
25	80	60	12	37.5	1.48	2.29	4.73
26	80	60	2	37.5	1.83	0.52	4.23
27	60	60	7	37.5	2.04	2.70	4.18
28	80	60	7	60.0	1.76	3.14	6.25
29	60	40	12	37.5	0.94	2.69	3.94

**Table 2:** ANOVA results for DPPH, FRAP, and TPC responses.

**DPPH**					
**Source**	**Sum of Squares**	**Df**	**Mean Square**	**F-Ratio**	***p*-Value**
A	0.974067	1	0.974067	17.50	0.0139 *
B	0.00345051	1	0.00345051	0.06	0.8156
C	0.662551	1	0.662551	11.90	0.0261 *
D	0.0425043	1	0.0425043	0.76	0.4315
AA	0.229943	1	0.229943	4.13	0.1119
AB	0.0555739	1	0.0555739	1.00	0.3743
AC	0.00159037	1	0.00159037	0.03	0.8740
AD	0.000544973	1	0.000544973	0.01	0.9259
BB	0.0934723	1	0.0934723	1.68	0.2648
BC	0.00276304	1	0.00276304	0.05	0.8346
BD	0.0388503	1	0.0388503	0.70	0.4505
CC	1.62797	1	1.62797	29.24	0.0057 **
CD	0.0000223956	1	0.0000223956	0.00	0.9850
DD	0.0504238	1	0.0504238	0.91	0.3951
Lack of fit	0.367673	10	0.0367673	0.66	0.7296
Pure error	0.222669	4	0.0556673		
Total (corr.)	4.06348	28			
R^2^	0.8547				
Adjusted R^2^	0.7094				
CV (%)	12.95				
**FRAP**					
**Source**	**Sum of Squares**	**Df**	**Mean Square**	**F-Ratio**	***p*-Value**
A	4.26738	1	4.26738	28.85	0.0058 **
B	0.324264	1	0.324264	2.19	0.2128
C	10.1047	1	10.1047	68.31	0.0012 **
D	0.136747	1	0.136747	0.92	0.3908
AA	0.493357	1	0.493357	3.34	0.1418
AB	0.0251601	1	0.0251601	0.17	0.7012
AC	0.24526	1	0.24526	1.66	0.2673
AD	0.50992	1	0.50992	3.45	0.1369
BB	0.0775534	1	0.0775534	0.52	0.5091
BC	0.377365	1	0.377365	2.55	0.1855
BD	0.00832178	1	0.00832178	0.06	0.8242
CC	3.05449	1	3.05449	20.65	0.0105 *
CD	0.0498219	1	0.0498219	0.34	0.5928
DD	0.94252	1	0.94252	6.37	0.0651
Lack of fit	4.08977	10	0.408977	2.76	0.1696
Pure error	0.591696	4	0.147924		
Total (corr.)	24.8943	28			
R^2^	0.8119				
Adjusted R^2^	0.6239				
CV (%)	16.68				
**TPC**					
**Source**	**Sum of Squares**	**Df**	**Mean Square**	**F-Ratio**	***p*-Value**
A	26.4646	1	26.4646	220.39	0.0001 ***
B	0.230731	1	0.230731	1.92	0.2380
C	0.000250063	1	0.000250063	0.00	0.9658
D	1.78016	1	1.78016	14.82	0.0183 *
AA	0.000145457	1	0.000145457	0.00	0.9739
AB	0.0114068	1	0.0114068	0.09	0.7733
AC	0.00629103	1	0.00629103	0.05	0.8302
AD	1.8682	1	1.8682	15.56	0.0169 *
BB	1.40154	1	1.40154	11.67	0.0269 *
BC	1.64864	1	1.64864	13.73	0.0207 *
BD	0.221111	1	0.221111	1.84	0.2463
CC	2.15403	1	2.15403	17.94	0.0133 *
CD	0.0669926	1	0.0669926	0.56	0.4966
DD	0.701689	1	0.701689	5.84	0.0730
Lack of fit	2.3358	10	0.23358	1.95	0.2726
Pure error	0.480317	4	0.120079		
Total (corr.)	38.5406	28			
R^2^	0.9269				
Adjusted R^2^	0.8539				
CV (%)	9.18				

A, B, C, and D represent extraction temperature, ethanol concentration, pH, and irradiation time, respectively. R^2^: coefficient of determination, CV: coefficients of variation. * Significant, *p* < 0.05. ** Very significant, *p* < 0.01. *** Highly significant, *p* < 0.001.

**Table 3:** Chemical characterization of ASE obtained under MAE optimal conditions (57 min, 80 °C, pH 8, and 70% (*v*/*v*) ethanol), expressed per gram of AS (DW).

Response	Content
Extraction yield (wt%)	35.2 ± 0.07
Flavonoids (mg CE g^−1^)	1.42 ± 0.05
Soluble proteins (mg BSA g^−1^)	0.43 ± 0.08
Total polysaccharides (mg glucose g^−1^)	1.59 ± 0.05
DPPH (mg TE g^−1^)	4.21 ± 0.44
DPPH RSA (%)	78 ± 7
DPPH IC_50_ (mg mL^−1^)	64.96 ± 1.17
FRAP (mg AAE acid g^−1^)	3.85 ± 0.54
TPC (mg GAE g^−1^)	6.59 ± 0.25
ABTS (mg TE g^−1^)	6.20 ± 0.48

## Data Availability

The data presented in this study are available on request from the corresponding author.
